# MIA SH3 Domain ER Export Factor 3 Deficiency Prevents Neointimal Formation by Restoring BAT-Like PVAT and Decreasing VSMC Proliferation and Migration

**DOI:** 10.3389/fendo.2021.748216

**Published:** 2021-11-10

**Authors:** Yu Lei, Jianfei Xu, Mengju Li, Ting Meng, Meihua Chen, Yongfeng Yang, Hongda Li, Tao Zhuang, Junli Zuo

**Affiliations:** ^1^ Department of Geriatrics and Geriatrics Center, Ruijin Hospital, Jiaotong University School of Medicine, Shanghai, China; ^2^ Department of Physiology and Pathophysiology, School of Basic Medical Sciences, Fudan University, Shanghai, China; ^3^ Key Laboratory of Arrhythmias of the Ministry of Education of China, Research Center for Translational Medicine, Shanghai East Hospital, Tongji University School of Medicine, Shanghai, China; ^4^ Department of Anesthesiology, Anting Hospital, Jiading District, Shanghai, China; ^5^ Jinshan Hospital Centre for Tumor Diagnosis and Therapy, Jinshan Hospital, Fudan University, Shanghai, China

**Keywords:** PVAT, MIA3, VSMCs, neointima, adipocytes

## Abstract

Abnormal proliferation and migration of vascular smooth muscle cells (VSMCs) and excessive accumulation of dysfunctional PVAT are hallmarks of pathogenesis after angioplasty. Recent genome-wide association studies reveal that single-nucleotide polymorphism (SNP) in MIA3 is associated with atherosclerosis-relevant VSMC phenotypes. However, the role of MIA3 in the vascular remodeling response to injury remains unknown. Here, we found that expression of MIA3 is increased in proliferative VSMCs and knockdown of MIA3 reduces VSMCs proliferation, migration, and inflammation, whereas MIA3 overexpression promoted VSMC migration and proliferation. Moreover, knockdown of MIA3 ameliorates femoral artery wire injury-induced neointimal hyperplasia and increases brown-like perivascular adipocytes. Collectively, the data suggest that MIA3 deficiency prevents neointimal formation by decreasing VSMC proliferation, migration, and inflammation and maintaining BAT-like perivascular adipocytes in PVAT during injury-induced vascular remodeling, which provide a potential therapeutic target for preventing neointimal hyperplasia in proliferative vascular diseases.

## Introduction

Coronary stents are routinely placed in the treatment of coronary artery disease (CAD). Though application of drug-eluting stents (DES) that release antiproliferative drugs, such as paclitaxel-eluting stents and sirolimus-eluting stents, has dramatically increased success rate compared to regular bare-metal stents (BMS), in-stent re-stenosis (ISR) remains to be problematic in coronary interventional treatment (1, 2).

The underlying molecular mechanism of in-stent neointimal formation has not been fully understood. VSMCs are the major cell types of the medial layer arteries and normally adopt a quiescent, contractile phenotype to regulate vascular tone. However, VSMCs retain phenotypic plasticity and can dedifferentiate into a proliferative, synthetic state, which associated with altered contractile marker expression such as smooth muscle myosin II (SM-myosin II), smoothelin, calponin, and smooth muscle-actin (3–5). Vascular injury caused by angioplasty, stenting, or bypass surgery triggers phenotypic switch of vascular smooth muscle cells (VSMCs) and subsequent abnormal proliferation and migration of VSMCs, leading to excessive formation of neointima, which contributes to occlusive vascular diseases such as atherosclerosis, intimal hyperplasia associated with restenosis, and vein graft stenosis (6–9). Therefore, unraveling the molecular mechanisms involved in regulating VSMC phenotypic switch, proliferation, and migration is a vital step toward understanding the pathology of restenosis.

Adipose tissues are present at multiple locations in the body. Most blood vessels are surrounded with adipose tissue, which is referred to as perivascular adipose tissue (PVAT). PVAT shows characteristics of beige adipose tissue (BeAT) in human and brown adipose tissue (BAT)-like in mice. PVAT is not always BAT in mice and humans. It depends on the anatomic location and environmental/metabolic context. While endovascular injury originates in the endothelium, its impact is felt throughout the blood vessel wall, including the adventitia and perivascular adipocytes (10). Phenotypic changes in PVAT after vascular injury promote release of adipocytokines that can regulate inflammation, VSMC proliferation, and neovascularization, thereby contributing to neointimal formation.

MIA3 (also named TANGO1) protein is localized to the endoplasmic reticulum exit site, where it loads cargo molecules, such as collagen VII, into COPII carriers to promote their secretion out of the endoplasmic reticulum (11, 12). Several large-scale meta-genome-wide association studies (GWAS) identified significant association between SNP rs17465637 in the MIA3/TANGO1 gene and CAD in the European ancestry populations and also Chinese populations (13, 14). A recent GAWS for 12 atherosclerosis-relevant phenotypes identified that the risk of single-nucleotide polymorphism (SNP) rs67180937 in the chromosome 1q41 locus was associated with lower VSMC MIA3 expression and lower proliferation (15). However, the role of MIA3 during neointimal formation is unknown.

Here, the present study showed that MIA3 was upregulated during SMC phenotypic modulation, which was induced by FBS administration. Conversely, the knockdown of MIA3 expression led to impaired proliferation and migration of SMCs, while MIA3 overexpression induced SMC proliferation. Knockdown of MIA3 ameliorates femoral artery wire injury-induced neointimal hyperplasia and increases brown-like perivascular adipocytes. This preliminary study provides new insights into the role and molecular mechanisms of MIA3 controlling function of VSMC and PVAT and identifies a novel potential target for suppression of neointimal formation.The authors declare that all supporting data are available within the article and its online supplementary file.

### Murine Model of Femoral Artery Wire Injury

Femoral artery wire injury was established in male C57BL/6 mice, as previously described (16). Briefly, mice were subjected to left femoral artery injury using a diameter angioplasty guidewire (0.35-mm diameter; Cook Inc, Bloomington, IN) under ketamine HCl (100 mg/kg, intraperitoneal injection) and xylazine HCl (10 mg/kg, intraperitoneal injection) anesthesia and aseptic conditions. Wire-injured femoral arteries were harvested at 28 days postsurgery, fixed with 4% paraformaldehyde, embedded in paraffin wax, and sectioned at 8-μm intervals for histology analysis. The cross-sections of the injured arteries were obtained at 500 to 1500 μm distant from the ligation at 100-μm intervals.

Sections were stained with hematoxylin and eosin (Servicebio, China) and Masson trichrome (Servicebio, China) stain kit and the images were acquired with Leica DM750 microscope. Five levels of hematoxylin and eosin staining images at 200-μm intervals were used for quantification of neointimal formation per mice. Measurements were made for lumen circumference, the internal elastic lamina circumference, and the circumference of the external elastic lamina. Measurements were quantified using ImageJ software (National Institutes of Health, Maryland, USA). The neointimal area was determined by subtracting the luminal area from the area bound by the internal elastic lamina. The media area was determined by subtracting the area bound by the internal elastic lamina from the area bound by the external elastic lamina. The intima-to-media ratio was determined by the intimal area divided by the medial area. Percentage of stenosis was calculated as the ratio of the intimal area to the area inside the original internal elastic lamina. Measurements were performed with the observer blinded to experimental group.

The animal procedures were performed in accordance with the Institutional Animal Care and Use of Laboratory Animals and were approved by the Animal Care Committee of Shanghai Jiao Tong University.

### Histology

The tissue sections were immersed in sodium citrate buffer (10 mM, pH 6.0) and heat retrieved for 20 min in a 100°C water bath to perform antigen retrieval. The slices were permeabilized and blocked in PBS-T (0.02% Triton X-100) with 1% goat serum for 1 h. Immunostaining was performed using the following antibodies diluted in PBS-T (0.02% Triton X-100) at 4°C overnight: anti-MIA3 (1:200, 17481-1-AP; WUHAN SANYING; China), Rabbit anti-Ki67 (1:200, Abcam, ab16667), Goat anti-Perilipin-1 (1:200, Abcam, ab61682), and Rabbit anti-UCP1 (1:200, Abcam, ab234430). Slides were mounted with Vectashield mounting medium containing DAPI (Vector Laboratories, Burlingame, CA, USA). After washing with PBS, sections were incubated with secondary antibodies (488 nm conjugated anti-rabbit or goat secondary antibody and 594 nm conjugated anti-rabbit) diluted in PBS-T (0.02% Triton X-100) for 1 h at room temperature. Following wash with PBS for three times, slides were mounted with vectashield mounting medium containing DAPI (Vector Laboratories, Burlingame, CA, USA) and imaged using fluorescent microscope.

### Cell Culture

Human aortic VSMCs were purchased from ATCC company (PCS-100-012, USA) and cultured in Vascular Smooth Muscle Cell Growth Kit (PCS-100-042, USA) supplemented with 10% FBS (Hyclone, USA).

### Knockdown of MIA3 *In Vitro*

The siRNA were purchased from Genepharma company and transfected into cells using Lipofectamine 2000 (Invitrogen, Carlsbad, CA) for knockdown of MIA3.The sequence of the MIA3 oligos was 5’-CCAGGUAGUUCAUGAAUAU-3’.

### Overexpression of MIA3 *In Vitro*

The human MIA3 full-length cDNA was subcloned into pCMV3 vector and transfected into cells using Lipofectamine 2000 (Invitrogen, Carlsbad, CA) for knockdown of MIA3. The plasmid is confirmed by full-length sequencing.

### Real-Time Quantitative–Polymerase Chain Reaction

Real-time polymerase chain reaction was performed according to our laboratory workflow. The expression of the involved genes was normalized to GAPDH, and experiment was repeated in triplicate. The primer sequences were listed as follows (forward, reverse): MIA3-homo: 5’-AAGTTCCAACAGATGAGACGGA-3’, 5’-GGTTCAGGTTCCCTTTCCTTAG-3’; α-SMA-homo: 5’-GAGAGGAGCAAAATCTGTCCG-3’, 5’-GGGGGAATTATCTTTCCTGGTCC-3’; Cyclin D1-homo: 5’-TGGAGCCCGTGAAAAAGAGC-3’, 5’-TCTCCTTCATCTTAGAGGCCAC-3’; IL-1β-homo: 5’-AGCTACGAATCTCCGACCAC-3’, 5’-CGTTATCCCATGTGTCGAAGAA-3’; IL18-homo: 5’-TCTTCATTGACCAAGGAAATCGG-3’, 5’-TCCGGGGTGCATTATCTCTAC-3’; CCL7-homo: 5’-TGCTCAGCCAGTTGGGATTA-3’, 5’-GGACAGTGGCTACTGGTGGT-3’; CxCL8-homo: 5’-TTTTGCCAAGGAGTGCTAAAGA-3’, 5’-AACCCTCTGCACCCAGTTTTC-3’; GAPDH-homo: 5’-CACCAGGGCTGCTTTTAACT-3’, 5’-TGGGATTTCCATTGATGACA-3’; MIA3-mus: 5’-GTGAGGATGAAGGTGACGA-3’, 5’-CTTGCTACCCTGAAGACGA-3’; GAPDH-mus: 5’-TGTTTCCTCGTCCCGTAGA-3’, 5’-ATCTCCACTTTGCCACTGC-3’.

### Western Blot Analysis

We used RIPA Lysis Buffer (P0013C; Beyotime Biotechnology, China) to extract protein from SMCs. Twenty micrograms of protein was loaded in 8% sodium dodecyl sulfate–polyacrylamide gel electrophoresis gel. Antibodies used were as follows: anti-MIA3 (1:1,000, 17481-1-AP; WUHAN SANYING; China), anti-α-SMA (1:1,000, ab7817; Abcam; USA), and anti-Cyclin D (1:1,000, ab16663; Abcam; USA). A horseradish peroxidase-conjugated goat anti−rabbit secondary antibody (1:1,000, A0277; Beyotime Biotechnology, China) was then added to the membranes at room temperature for 2 h. Subsequently, ImageJ analysis software was used to quantify the bands of Western blot images, and glyceraldehyde-3-phosphate dehydrogenase (GAPDH) was used as internal reference. Each experiment was repeated three times.

### Immunofluorescence Staining

Cells were seeded on glass coverslips placed in 24-well plates and fixed with 4% paraformaldehyde for 15 min and then permeabilized with 0.25% Triton X-100 in PBS for 10 min. Then, cells were washed three times with PBS and blocked with 5% goat serum in PBS for 30 min. Next, the primary antibodies were used to incubate VSMCs overnight at 4°C. Appropriate secondary antibodies were incubated with VSMCs for 1 h at room temperature. DAPI was used to stain the nuclei. The images were acquired using a fluorescent microscope (Zeiss LSM780, Carl Zeiss).

### Cell Proliferation Assay

Cell viability was measured using the cell counting, MTT assay, and EdU staining assay. In the cell counting assay, VSMCs transfected with MIA3 siRNA or overexpressing vector and scramble siRNA or control empty vector were seeded at an initial density of 2 × 10^4^ per well in a 12-well plate in VSMC culture medium with 10% FBS, and the cells were harvested and counted at the designated time points. For MTT assay, VSMCs were seeded in a 96-well plate in VSMC culture medium with 10% FBS, and 50 μl of MTT solution [5 mg/ml; A600799, Sangon Biotech (Shanghai) Co., Ltd.] was added to each well and incubated for 4 h at the designated time points. To dissolve the formazan crystals, 80 μl of mixture of 40 ml of isopropanol plus 44 μl of 37% HCl was added to each well after the media were removed. Absorbance was measured at 570 nm using a microplate reader (Bio-Rad). EdU staining assay was performed using the EdU Staining Proliferation Kit (iFluor 647) according to the protocol booklet. Briefly, equal VSMCs were seeded in a six-well plate after starved for 24 h and allowed to grow in VSMC with 10% FBS culture medium, and the EdU solution was added to VSMCs for 2 h under growth conditions. VSMCs were fixed in 4% paraformaldehyde, permeated, and stained with reaction mix to fluorescently label EdU for 30 min. The samples were mounted on glass slides and were visualized using an inverted fluorescent microscope (Carl Zeiss, Oberkochen, Germany).

For propidium iodide cell cycle analysis, VSMCs were starved for 24 h and grown in culture medium for 24 h. VSMCs were then harvested and immersed in 70% ethanol at the designated time point. Cell DNA was stained with 50 μg/ml propidium iodide and 20 μg/ml RNase at 37°C for 30 min. Cell cycle was analyzed using a flow cytometry with a FACS canTM flow cytometer (BD Biosciences, Mansfield, MA, USA).

### Cell Migration Assay

For the wound healing assay, VSMCs (2 × 10^5^ cells) transfected with MIA3 siRNA or overexpressing vector and scramble siRNA or control empty vector were plated onto six-well plates and serum-starved for 24 h. An artificial wound (scratch) was generated using a 200-μl pipette tip and cultured for 24 h in serum-starved medium. SMCs were visualized using a microscope and captured images were assessed using Image−Pro Plus software.

For the Transwell migration assay, VSMCs were cultured on the microporous membrane (8.0 µm) in the upper chamber of the Transwell (Costar 3422; Corning Incorporated, NY, USA) in serum-free medium for 12 h, and VSMC culture medium with 10% FBS was added into the lower chamber. After 24 h, SMCs were allowed to migrate from the upper chamber to the underside of the membrane. The unmigrated cells in the upper chamber were gently removed using a cotton swab. Cells migrated through the membrane to the lower chamber were fixed in paraformaldehyde and stained with 0.05% crystal violet. Migrated SMCs on the lower membrane were counted using an Olympus light microscope and analyzed using the Image J software.

### Lentivirus Generation and Transduction

Lentivirus was produced using pLV10N-U6-shRNA vector and mouse MIA3 shRNA was cloned into the vector, and generated in the 293T viral packaging cell line. Femoral arteries were transduced locally with 10^9^ IU per mouse in the presence of 10 μg/ml DEAE-dextran after wire injury. The efficiency of MIA3 knockdown was estimated by real-time quantitative-polymerase chain reaction. The sequence of MIA3 shRNA was GCAACCAGACTGGTCACTTCA.

### RNA Extraction and RNA High-Throughput Sequencing

RNA-sequencing was conducted on MIA3 knockdown and control scramble human aortic smooth muscle cells (HASMCs). Total RNA was extracted using TRIzol (Invitrogen, USA) following the manufacturer’s instructions. The total RNA concentration, the RIN value, 28S/18S, and the fragment size were measured using an Agilent 2100 Bioanalyzer (Agilent, USA). Oligo(dT)-attached magnetic beads were used to purify mRNA. The BGISEQ-500 (Shenzhen Huada Gene, China) platform was used for high-throughput sequencing to obtain a 50-bp sequencing read. The raw data were subjected to quality control to obtain effective reads. SOAPnuke (v 1.5.2) and Trimmomatic (v0.36) were used to perform statistical analysis and filter out reads of low to moderate quality, polluted connectors, and unknown nucleotides with high N content before data analysis to ensure reliability. The clean reads were mapped to the reference genome using HISAT2 (v2.0.4). Ericscript (v0.5.5) and rMATS (V3.2.5) were used to fusion genes and differential splicing genes (DSGs).

### Statistical Analysis

All values in the graphs represent the mean ± SEM. Comparison between two groups were compared using an unpaired Student’s *t*-test. Statistical analyses were performed in SPSS version 13.0 (SPSS, Inc., Chicago, IL). *p* < 0.05 was considered to indicate a statistically significant difference.

## Results

### MIA3 Expression Is Increased in Proliferative Vascular Smooth Muscle Cells

**Figure 1:**
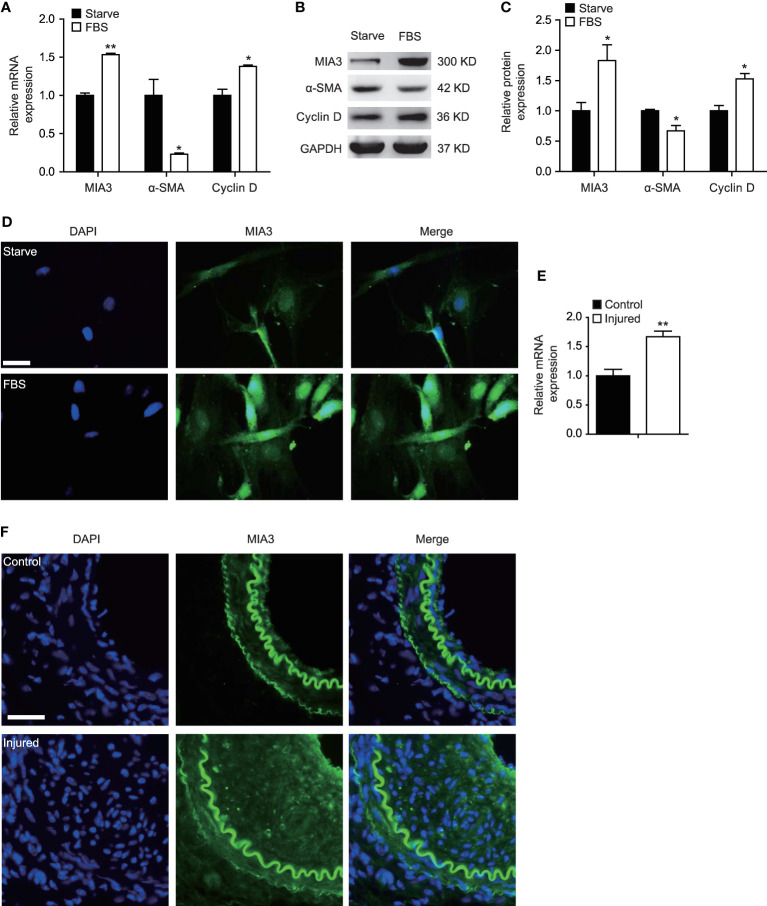
MIA3 expression is increased in proliferative vascular smooth muscle cells (VSMCs). **(A)** mRNA levels of MIA3, α-SMA (smooth muscle α-actin), and Cyclin D1 by quantitative real-time reverse transcription polymerase chain reaction in VSMCs after treatment with medium containing 10% fetal bovine serum for 24 h and the control FBS-starve VSMCs. Data are represented as the mean ± SEM, *n* = 3. *p < 0.05, **p < 0.01, *t*-test. **(B)** At 48 h after FBS treatment, VSMC extracts were collected for determining the protein levels of MIA3, α-SMA (smooth muscle α-actin) and Cyclin D1 by Western blotting. GAPDH served as loading control expression. **(C)** Quantification of protein expression normalized to the levels of GAPDH. Data are represented as the mean ± SEM, *n* = 3. *p < 0.05, *t*-test. **(D)** Immunofluorescent staining of MIA3 in VSMCs after treatment with medium containing 10% fetal bovine serum for 24 h and the control FBS-starve VSMCs. DAPI represents indicates 4’,6-diamidino-2-phenylindole throughout the article; scale bar: 50 μm. **(E)** mRNA levels of MIA3 by quantitative real-time reserve transcription polymerase chain reaction in femoral artery after wire injury for 28 days. Data are represented as the mean ± SEM, *n* = 3. **p < 0.01, *t*-test. **(F)** Immunofluorescent staining of MIA3 in the femoral artery following wire injury for 28 days; scale bar: 100 μm.

FBS is a potent mediator of the SMC phenotypic modulation from a contractile to a synthetic state by promoting SMC proliferation as well as repressing SMC marker gene expression. To investigate whether MIA3 is associated with VSMC proliferation, we treated the cultured HASMC with FBS, and our data revealed that MIA3 is expressed in VSMCs and the mRNA and protein level of MIA3 in cultured proliferating HASMCs are significantly increased compared with the serum-starved quiescent HASMCs, whereas SMC contractile gene α-SMA was significantly reduced (**Figures 1A–C**). Proliferation gene Cyclin D expression was enhanced in parallel with MIA3 expression. Immunofluorescence staining showed that the expression of MIA3 in cultured HASMCs was induced by FBS (**Figure 1D**). These data suggest that upregulation of MIA3 is positively correlated with the synthetic SMC phenotype and MIA3 may regulate VSMC proliferation.

As FBS-induced VSMC proliferation contributes to neointimal hyperplasia, we detected MIA3 expression changes during neointimal formation in a mouse femoral artery wire injury model. Compared with the control sham group, MIA3 mRNA level in femoral arteries at 28 days after wire injury was markedly increased (**Figure 1E**). Consistent with quantitative real-time reserve transcription polymerase chain reaction, MIA3 protein expression was increased in the femoral arteries at 28 days after wire injury by immunofluorescent staining (**Figure 1F**). Taken together, our results demonstrate that the increase of MIA3 in the femoral arteries after wire injury may be involved in neointimal hyperplasia

### Knockdown of MIA3 Inhibits Vascular Smooth Muscle Cell Proliferation *In Vitro*

**Figure 2:**
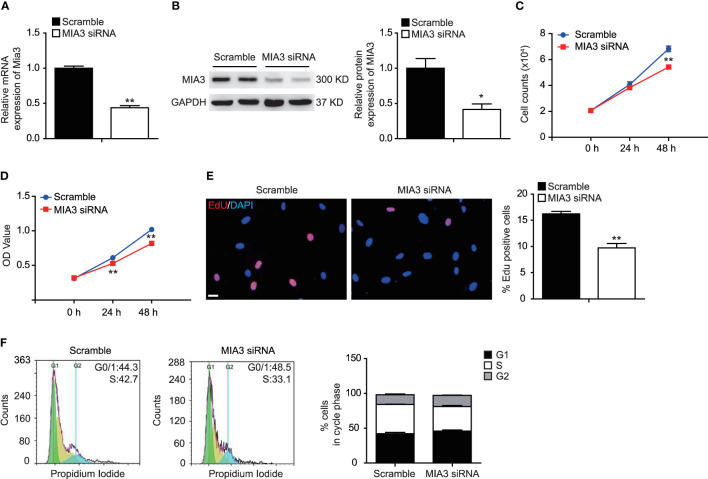
Knockdown of MIA3 inhibits vascular smooth muscle cell (VSMC) proliferation. **(A)** MIA3 mRNA levels in human aortic smooth muscle cells (HASMCs) with small interfering RNA (siRNA) MIA3 and scramble siRNA. Data are represented as the mean ± SEM, *n* = 3. **p < 0.01, *t*-test. **(B)** MIA3/TANGO1 protein levels in human aortic smooth muscle cells (HASMCs) with small interfering RNA (siRNA) MIA3 and scramble siRNA with quantitative data at right. GAPDH served as loading control expression. Data are represented as the mean ± SEM, *n* = 3. *p < 0.05, *t*-test. **(C, D)** VSMC proliferation was determined by cell counts **(C)** and 3-[4,5-dimethylthiazol-2-yl]-2,5 diphenyl tetrazolium bromide (MTT) assay **(D)** in VSMCs with or without transfection of MIA3-specific siRNAs. All data were indicated as the means ± SEM, *n* = 3. **p < 0.01, *t*-test. **(E)** VSMC proliferation was determined by 5-ethynyl-2’deoxyuridine (EdU) incorporation in VSMCs with or without transfection of MIA3-specific siRNAs. Percentage of EdU staining-positive cells was quantified on the right. Scale bar: 50 μm. Data are represented as the mean ± SEM, *n* = 3. **p < 0.01, *t*-test. **(F)** VSMC proliferation was determined by PI-cell cycle analysis in VSMCs with or without transfection of MIA3/TANGO1-specific siRNAs. Data are represented as the mean ± SEM, *n* = 3, *t-test*.

Proliferation of VSMCs plays a vital role in the development of neointimal formation. To investigate whether MIA3 participated in the proliferation of VSMCs, we used siRNA targeting MIA3 to silence its expression in VSMCs. As shown in **Figures 2A, B**, significantly reduced expression of MIA3 was observed in HASMCs infected with MIA3 siRNA compared with control scramble siRNA. As expected, cell counting, MTT assay, and EdU incorporation assay showed that MIA3 knockdown attenuated VSMC proliferation (**Figures 2C–E**). Furthermore, flow cytometry analysis of cell cycle status showed that MIA3 knockdown caused a significant increase in the percentage of cells in G1 phase (from 42.1% ± 2.0% to 46.1% ± 1.5% in FBS-stimulated cells) but decreased the percentage of cells in S phase (from 42.2% ± 0.4% to 35.1% ± 1.6% in FBS-stimulated cells) (**Figure 2F**). Collectively, these results demonstrate that knockdown of MIA3 attenuates VSMC proliferation *in vitro*.

### Knockdown of MIA3 Inhibits Vascular Smooth Muscle Cell Migration *In Vitro*

**Figure 3:**
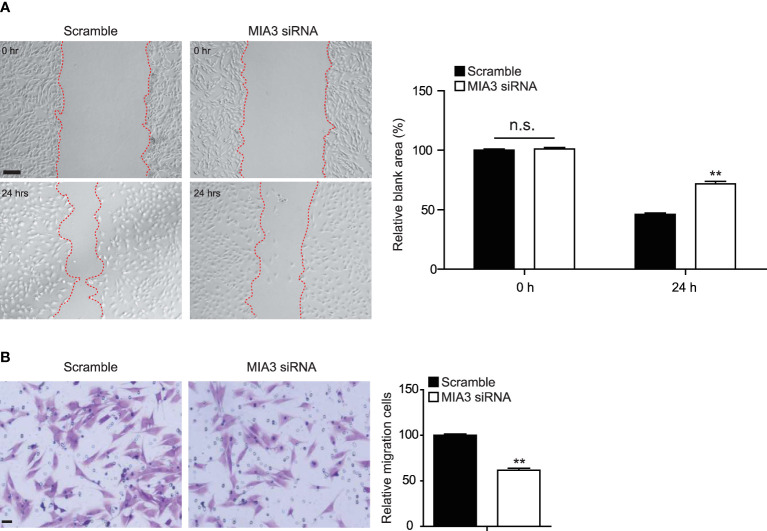
Knockdown of MIA3 inhibits vascular smooth muscle cell (VSMC) migration. **(A)** The wound-induced cell migration assay was performed in VSMCs with or without transfection of MIA3-specific small interfering RNA. The relative blank wound areas in the left were quantified on the right. Scale bar: 100 μm. Data are represented as the mean ± SEM, *n* = 3. n.s. indicates nonsignificant. **p < 0.01, *t*-test. **(B)** The transwell assay in VSMCs with MIA3 siRNA or scramble siRNA. The transferred and stained cells were counted on the right. Scale bar: 50 μm. Data are represented as the mean ± SEM, *n* = 3. *t*-test.

VSMC migration from the medial layer is another key event to build neointima. Wound healing and Boyden chamber assay were used to test the role of MIA3 in the VSMC migration. Consistent with the role of MIA3 in VSMC proliferation, MIA3-specific siRNA efficiently attenuated VSMC migration by wound healing (**Figure 3A**) and Boyden chamber assay (**Figure 3B**). The above data suggested that MIA3 knockdown in VSMC decreases cell migration *in vitro*.

### Overexpression of MIA3 Promotes Vascular Smooth Muscle Cell Proliferation and Migration *In Vitro*

**Figure 4:**
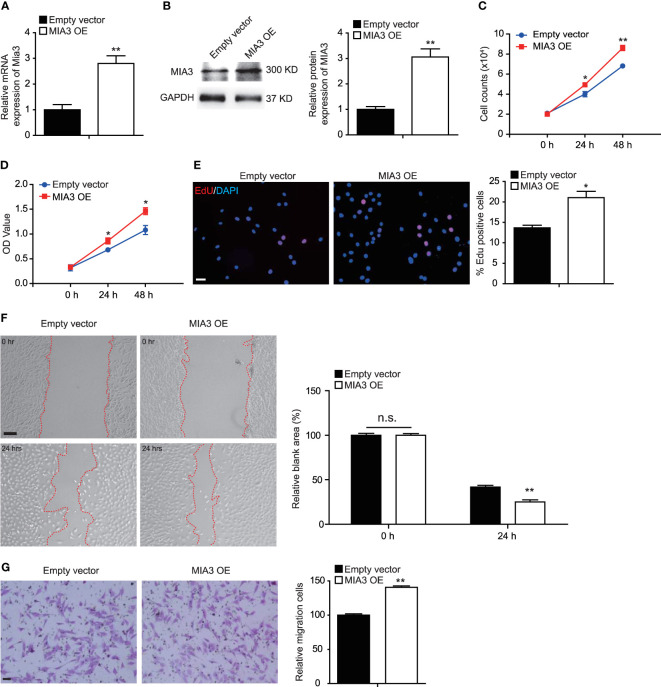
MIA3 overexpression promotes vascular smooth muscle cell (VSMC) proliferation and migration. **(A)** MIA3 mRNA levels in human aortic smooth muscle cells (HASMCs) transfected with MIA3 overexpressing vector (OE) and control empty vector. Data are represented as the mean ± SEM, *n* = 3. **p < 0.01, *t*-test. **(B)** MIA3 protein levels in human aortic smooth muscle cells (HASMCs) transfected with MIA3 overexpressing vector and control empty vector with quantitative data on the right. GAPDH served as loading control expression. Data are represented as the mean ± SEM, *n* = 3. *p < 0.05, *t*-test. **(C, D)** VSMC proliferation was determined by cell counts **(C)** and 3-[4,5-dimethylthiazol-2-yl]-2,5 diphenyl tetrazolium bromide (MTT) assay **(D)** in VSMCs with or without transfection of MIA3 overexpressing vector. All data were indicated as the means ± SEM, *n* = 3. **p < 0.01, *t*-test. **(E)** VSMC proliferation was determined by 5-ethynyl-2’-deoxyuridine (EdU) incorporation in VSMCs with or without transfection of MIA3 overexpressing vector. Percentage of EdU staining–positive cells was quantified on the right. Scale bar: 50 μm. Data are represented as the mean ± SEM, *n* = 3. **p < 0.01, *t*-test. **(F)** The wound-induced cell migration assay was performed in VSMCs with or without transfection of MIA3 overexpressing vector. The relative blank wound areas in the left were quantified on the right. Scale bar: 100 μm. Data are represented as the mean ± SEM, *n* = 3. n.s. indicates nonsignificant. **p < 0.01, *t*-test. **(G)** The transwell assay in VSMCs transfected with MIA3 overexpressing vector or empty vector. The transferred and stained cells were counted on the right. Scale bar: 50 μm. Data are represented as the mean ± SEM, *n* = 3. **p < 0.01, *t*-test.

Our above data showed that knockdown of MIA3 blocked VSMC proliferation and migration. We next investigated the effect of MIA3 overexpression on VSMC proliferation and migration. The efficiency of Foxp1 overexpression was confirmed by RT-qPCR and Western blot (**Figures 4A, B**). Importantly, VSMC proliferation was increased upon overexpression of MIA3 determined by cell counting, MTT assay, and EdU incorporation assay (**Figures 4C**–**E**). The wound healing and Boyden chamber assay further demonstrated that MIA3 overexpression increased VSMC migration (**Figures 4F, G**). Together, upregulation of MIA3 itself promotes cell proliferation and migration of VSMCs.

### Knockdown of MIA3 Attenuates Femoral Artery Wire Injury–Induced Neointimal Formation in Mice

**Figure 5:**
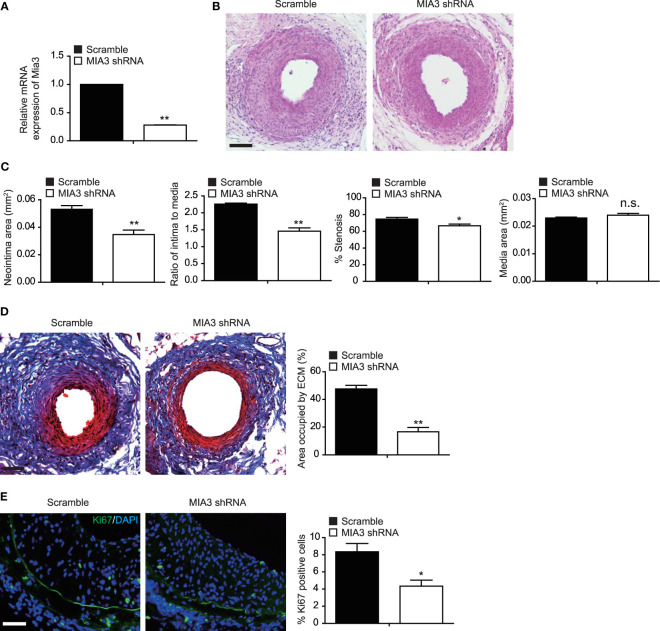
Knockdown MIA3 ameliorates femoral artery wire injury-induced neointimal hyperplasia in mice. **(A)** Quantitative real-time reserve transcription polymerase chain reaction was performed to confirm the decreased expression of MIA3 in the injured femoral arteries. Data are represented as the mean ± SEM, *n* = 5. **p < 0.01. *t*-test. **(B)** Representative hematoxylin and eosin (H&E) staining of mouse femoral arteries after wire injury for 28 days from mice infected with MIA3 shRNA lentivirus and scramble shRNA lentivirus. Scale bar: 100 μm. **(C)** The neointima area, intima-to-media ratio, % stenosis, and media area of wire-injured femoral arteries from mice infected with MIA3 shRNA lentivirus and scramble shRNA lentivirus. *p < 0.05, **p < 0.01, n.s. indicates nonsignificant. *n* = 5, *t*-test. **(D)** Representative Masson trichrome staining of mouse femoral arteries after wire injury for 28 days from mice infected with MIA3 shRNA lentivirus and scramble shRNA lentivirus, with quantification data on the right. Scale bar: 100 μm. Data are represented as the mean ± SEM, *n* = 5. **p < 0.01. *t*-test. **(E)** Knockdown MIA3 with shRNA lentivirus exhibit a significant reduction of Ki67-positive cells in the neointima at 28 days after femoral artery wire injury, with representative images (left) and quantification data (right; *n* = 5 for each group). Data are represented as the mean ± SEM, *n* = 5. *p < 0.05. *t*-test.

To investigate the function of MIA3 in femoral artery wire injury–induced neointimal formation in mice, lentiviral control shRNA or lentiviral MIA3 shRNA was perivascularly applied to femoral arteries immediately after wire injury, as described in the previous studies (16, 17). The decrease of MIA3 expression in wire-injured femoral artery applied with lentiviral MIA3 shRNA was confirmed by real-time quantitative–polymerase chain reaction (**Figure 5A**). Consequently, at 28 days after femoral artery wire injury, knockdown of MIA3 significantly attenuated neointimal formation, as shown by the decreased neointimal area, neointima/media ratio, and percentage of stenosis in the lentiviral MIA3 shRNA-treated injured artery, but has no influence on vessel media area (**Figures 5B, C**). Masson trichrome staining showed that knockdown of MIA3 significantly reduced ECM deposition after vascular injury (**Figure 5D**). Immunostaining showed a significant reduction of Ki67-positive cells (**Figure 5E**) in the neointima of MIA3 knockdown mice, suggesting that knockdown of MIA3 inhibits the cell proliferation contributing to decreased neointimal hyperplasia after wire injury of femoral artery. These results indicate that MIA3 knockdown markedly suppresses neointimal formation in mice.

### Knockdown of MIA3 Maintains BAT-Like Perivascular Adipocytes in PVAT

**Figure 6:**
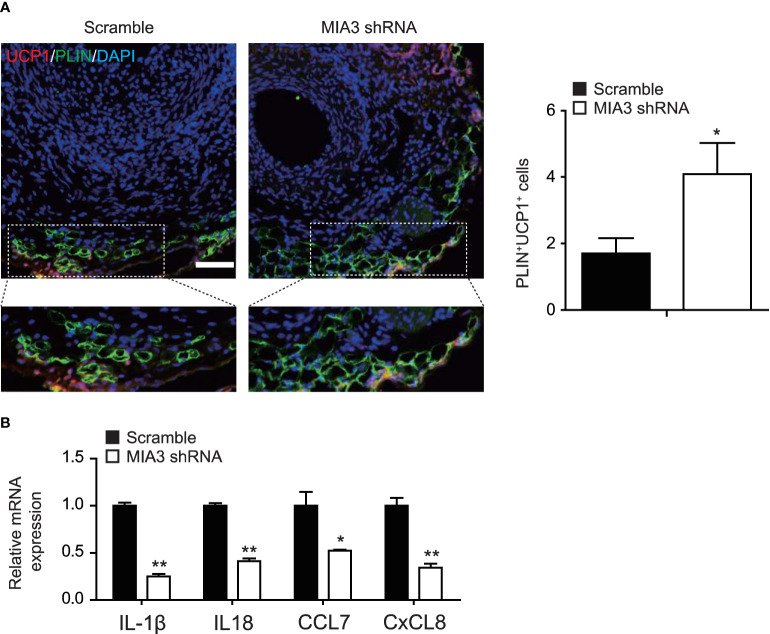
Knockdown MIA3 increases the expression of uncoupling protein-1 in the perivascular adipocytes. **(A)** Knockdown MIA3 with shRNA lentivirus exhibited significant increase of PLIN and UCP-1 double-positive cells in the neointima at 28 days after femoral artery wire injury, with representative images (left) and quantification data (right; *n* = 5 for each group). Data are represented as the mean ± SEM. *p < 0.05. *t*-test. **(B)** Knockdown MIA3 with siRNA in VSMCs significantly decreased expression of IL-1β, IL18, CCL7, and CxCL8 by quantitative real-time reserve transcription polymerase chain reaction. Data are represented as the mean ± SEM, *n* = 3. *p < 0.05, **p < 0.01, *t*-test.

Extensive inflammatory cell infiltration around the vasculature in response to vascular injury induces histological and phenotypic changes of perivascular adipocytes. Dysfunctional PVAT secretes disease-promoting factors that exacerbate pathogenesis of neointimal formation. So, we investigated whether MIA3 deficiency altered PVAT features from BAT-like to WAT-like contributing to neointimal formation. Immunostaining showed a significant increase of UCP-1-positive cells (**Figure 6A**) in the neointima of MIA3 knockdown mice, suggesting that MIA3 deficiency increased brown-like perivascular adipocytes.

PVAT dysfunction is characterized by its inflammatory character, and VSMCs are a significant source of chemokines and cytokines (18). To investigate the mechanism of MIA3 in maintaining BAT-like perivascular adipocytes in PVAT, we performed RNA-sequencing of MIA3 shRNA-treated VSMC and scramble VSMC, and found that knockdown MIA3 in VSMC decreased IL-1β, IL18, CCL7, and CxCL8 expression (Online uploaded excel file), which were confirmed by quantitative real-time reserve transcription polymerase chain reaction (**Figure 6B**). The above results illustrate that knockdown MIA3 in VSMC maintains BAT-like perivascular adipocytes in PVAT *via* inhibiting expression of inflammatory factors.

## Discussion

Here, we discovered that MIA3 is a novel regulator in promoting VSMC proliferation and migration during neointimal formation. Knockdown of MIA3 reduces proliferation and migration of SMC. In contrast, MIA3 overexpression promoted VSMC migration and proliferation. Furthermore, administration of MIA3 shRNA lenti-virus attenuated femoral artery wire injury-induced neointima in mice. In addition, knockdown MIA3 maintains BAT-like perivascular adipocytes in PVAT *via* inhibiting expression of inflammatory factors following femoral artery wire injury. Our findings identified MIA3 as a novel target for developing antineointima drugs during vascular repair.

VSMCs are the major cell types of medial layer arteries and play a pivotal role in regulating the remodeling process of the vessel wall (19). Fully differentiated SMCs are almost quiescent with little proliferation and are programmed for contraction with relatively high expression of SM myosin heavy chain (SMMHC), SM22a, and calponin. However, in response to local vascular injury, SMCs dedifferentiate from contractile phenotype toward a synthetic state, which was characterized by a decreased expression of contractile SMC marker genes and increased rates of migration and proliferation (19, 20). Subsequent excess proliferation and migration result in an accumulation of synthetic SMCs in the stented artery, which contributes to the in-stent restenosis. Thus, inhibiting the proliferation and migration of intravascular SMC is the predominant therapeutic strategy to prevent the excessive formation of neointima (21, 22).

MIA SH3 Domain ER Export Factor 3 (MIA3) is an evolutionarily conserved endoplasmic reticulum-resident transmembrane protein and is required for the export of collagen VlI (COL7A1) from the endoplasmic reticulum. Mice lacking MIA3 are defective for the secretion of numerous collagens, including collagens I, II, III, IV, VII, and IX, from chondrocytes, fibroblasts, endothelial cells, and mural cells (23). Our study demonstrated that knockdown of MIA3 significantly reduced ECM deposition after vascular injury (**Figure 5D**), indicating that MIA3 may regulate vascular remodeling in response to injury through regulating extracellular matrix secretion.

Genome-wide association studies (GWAS) have described an association between MIA3 rs17465637 A/C polymorphisms and CAD and myocardial infarction (24–26). A recent study observed a significant reduction of MIA3 protein in VSMCs in thin fibrous caps of late-stage atherosclerotic plaques compared to early fibroatheroma with thick and protective fibrous caps in mice and humans (15), indicating that high MIA3 expression may promote atheroprotective VSMC phenotypic transitions. However, the detailed role of MIA3 in VSMC phenotypic transitions is unclear. FBS is a key stimulus for VSMC proliferation, migration, and phenotypic switch contributing to neointimal formation (27). In this study, the increase of MIA3 in FBS-induced VSMC may contribute to the development of injury-induced neointimal formation. The knockdown of MIA3 results indicated that VSMC proliferation and migration, which are the critical cellular events in vascular neointimal lesion formation, were regulated, at least in part, by MIA3. We showed that local transfer of lentiviral MIA3 shRNA onto the injured arteries could significantly reduce VSMC proliferation and decreased neointimal formation at the 28th day post-injury, providing a basis for preventing or inhibiting in-stent restenosis *via* MIA3 siRNA-coated stents.

A previous study demonstrated that overexpression of endothelial MIA3 significantly increased EC proliferation, migration, and EC tube formation, which suggest that EC-MIA3 might alleviate neointimal formation (24). We will pursue the role and mechanism of endothelial MIA3 in vascular injury neointimal formation in the future.

PVAT surrounds most large blood vessels and plays important roles in vascular homeostasis and excessive accumulation of dysfunctional PVAT leads to vascular disorders by targeting VSMCs and endothelial cells (10, 28). PVAT displays heterogeneity according to species and locations. Reversing the white features of PVAT to brown characteristics or maintaining PVAT beige features might be a crucial strategy to maintain a healthy vasculature (28). Our study revealed that MIA3 deficiency increased the expression of uncoupling protein-1 (UCP-1), the brown fat marker, in perivascular adipocytes, which indicated that MIA3 deficiency in VSMCs reversed the white features of PVAT to brown characteristics. RNA-sequencing bioinformatic analysis indicated 694 upregulated genes and 628 downregulated genes in MIA3-shRNA- and scramble-shRNA-treated VSMCs (Online uploaded excel file). The differentially expressed genes between scramble and MIA3 knockdown VSMCs were enriched for protein processing in endoplasmic reticulum, Hippo signaling pathway, TGF-β signaling pathway, etc. Knockdown MIA3 in VSMC decreased IL-1β, IL18, CCL7, and CxCL8 expression according to the RNA-sequencing bioinformatic analysis, which implies the important roles of inflammatory factors derived from VSMCs in injury-induced neointimal formation.

In summary, we present compelling evidence that MIA3 deficiency in VSMCs prevents neointimal formation by decreasing VSMC proliferation and migration and restoring BAT-like PVAT during injury-induced vascular remodeling. Our study found for the first time that inhibition of MIA3 in the injured arteries can prevent postangioplasty restenosis, supporting a potential role for MIA3 and its target genes in a variety of proliferative vascular diseases. These findings may have extensive implications for the treatment of occlusive vascular diseases.

## Data Availability Statement

The data presented in the study is deposited in the GEO repository, accession number GSE186951.

## Ethics Statement

The animal procedures were performed in accordance with the Institutional Animal Care and Use of Laboratory Animals and were approved by the Animal Care Committee of Shanghai Jiao Tong University.

## Author Contributions

JZ conceived and designed the study. TZ organized and supervised the study. YL and JX conducted the experiments of this study. ML, TM, MC, YY, and HL conducted the statistical analysis. JZ and TZ drafted the article. All authors contributed to the article and approved the submitted version.

## Funding

This study was funded by the National Natural Science Foundation of China (Grant Nos. 82070456 and 81800253), the Shanghai Municipal Hospital New Frontier Technology Joint Project (Grant no. SHDC12019X20), the Shanghai Municipal Commission of Health and Family Planning (Grant no. 2020YJZX0124), and Shanghai Rising-Star Program (Grant no. 21QA1401400).

## Conflict of Interest

The authors declare that the research was conducted in the absence of any commercial or financial relationships that could be construed as a potential conflict of interest.

## Publisher’s Note

All claims expressed in this article are solely those of the authors and do not necessarily represent those of their affiliated organizations, or those of the publisher, the editors and the reviewers. Any product that may be evaluated in this article, or claim that may be made by its manufacturer, is not guaranteed or endorsed by the publisher.

## Supplementary Material

The Supplementary Material for this article can be found online at: https://www.frontiersin.org/articles/10.3389/fendo.2021.748216/full#supplementary-material
